# Reachability analysis of FMI models using data-driven dynamic sensitivity

**DOI:** 10.1177/00375497241261409

**Published:** 2024-07-25

**Authors:** Sergiy Bogomolov, Cláudio Gomes, Carlos Isasa, Sadegh Soudjani, Paulius Stankaitis, Thomas Wright

**Affiliations:** 1 School of Computing, Newcastle University, UK; 2 Department of Electrical and Computer Engineering, Aarhus University, Denmark; 3 Max Planck Institute for Software Systems, Germany; 4 Department of Computing Science and Mathematics, University of Stirling, UK

**Keywords:** Reachability analysis, digital twins, Functional Mock-up Interface, dynamic sensitivity equations, Lipschitz constant

## Abstract

Digital twin is a technology that facilitates a real-time coupling of a cyber–physical system and its virtual representation. The technology is applicable to a variety of domains and facilitates more intelligent and dependable system design and operation, but it relies heavily on the existence of digital models that can be depended upon. In realistic systems, there is no single monolithic digital model of the system. Instead, the system is broken into subsystems, with models exported from different tools corresponding to each subsystem. In this paper, we focus on techniques that can be used for a black-box model, such as the ones implementing the Functional Mock-up Interface (FMI) standard, formal analysis, and verification. We propose two techniques for simulation-based reachability analysis of models. The first one is based on system dynamics, while the second one utilizes dynamic sensitivity analysis to improve the quality of the results. Our techniques employ simulations to obtain the model’s sensitivity with respect to the initial state (or model’s Lipschitz constant) which is then used to compute reachable states of the system. The approaches also provide probabilistic guarantees on the accuracy of the computed reachable sets that are based on simulations. Each technique requires different levels of information about the black-box system, allowing the readers to select the best technique according to the capabilities of the models. The validation experiments have demonstrated that our proposed algorithms compute accurate reachable sets of stable and unstable linear systems. The approach based on dynamic sensitivity provides an accurate and, with respect to system dimensions, more scalable approach, while the sampling-based method allows a flexible trade-off between accuracy and runtime cost. The validation results also show that our approaches are promising even when applied to nonlinear systems, especially, when applied to larger and more complex systems. The reproducibility package with code and data can be found at https://github.com/twright/FMI-Reachability-Reproducibility.

## 1. Introduction

Digital twins (DTs) are an emerging technology that makes it possible to monitor, optimize, and control cyber–physical assets using their virtual representation (kept as a mirror of reality) in real-time.^1^ They provide critical services such as state estimation, visualization, what-if analysis, anomaly detection, and self-adaptation.

Because DT services rely heavily on the existence of models of the cyber–physical systems^2,3^ (CPS), the dependability of the DT is a direct consequence of how much we can depend upon the models’ simulation. For example, prior to adapting the controller of the CPS, the DT needs to find the optimal and safe configuration by, for example, running simulations with alternative configurations on future predicted scenarios, while checking that safety properties are satisfied. If there is uncertainty in the model parameters, as there often is in continuous and hybrid system models whose parameters are identified from sensor data, then we may be interested in computing bounds that enclose all simulation results, based on the possible parameter values, in a technique called reachability analysis. An introduction and survey of the topic of reachability analysis are provided in the study by Althoff et al.^4^ and an example application for DTs is presented in the study by Wright et al.^5^

To compute reachable states of the system generally requires knowing a model of the system, which for CPSs can be hard to obtain or even unavailable because of the myriad of modeling and simulation tools used in engineering practice. Fortunately, the industry has formulated standards that make it possible to represent and integrate black box, IP-protected models. One such standard is the Functional Mock-up Interface (FMI),^6^ which is currently supported by more than 150 tools. Because of these reasons, in this paper, we focus on a class of reachability analysis techniques that are data-driven (i.e. they rely on data generated from simulations), which can be applied to black-box models. Although several data-driven reachability analysis approaches have been proposed in the literature, they either do not provide probabilistic guarantees on the completeness of the exploration, or discuss handle coupled models.

### 1.1. Contribution

In this paper, we build upon our previous work^7^ and propose a new method for computing reachable states of black-box coupled models. This reachability analysis method leverages advanced FMI standard functionality for retrieving partial derivatives of Functional Mock-up Unit (FMU) variables and numerical differential system solvers to solve dynamic sensitivity equations, which describe system sensitivity to changes in their initial conditions. The computed maximum sensitivity provides a scaling factor, which together with a nominal initial state space trajectory is used to compute approximate reachable sets.

In summary, the novel contributions of this paper are: (1) a dynamic sensitivity-based reachability analysis method of black-box models and (2) a method for composing dynamic sensitivity equation systems from coupled models implementing the FMI standard. The paper evaluates the new approach against our previously introduced data-driven method^7^ by comparing reachable sets computed for linear and nonlinear dynamical systems. We also validate our approaches against a leading model-based reachability analysis tool—Flow*.^8^

The paper is structured as follows. The following “Related work” section discusses related work and positions our reachability analysis approach. After that, our paper describes the preliminaries and the problem statement of the paper. The main contributions of the paper are presented in the “Reachability algorithms” section in which we formally describe our proposed reachability analysis and dynamic sensitivity equation composition algorithms. The “Validation experiments” section describes results obtained from comparing and validating algorithms, as well as discusses limitations and recommendations of the proposed methods. In the final section, we summarize our findings and propose directions for future work.

## 2. Related work

This paper extends our previous work,^7^ where we proposed a data-driven method for computing the reachable states of black-box models with probabilistic accuracy guarantees, given a sufficient number of samples is used. This reachability method was based on estimating a maximum Lipschitz constant by simulating a model from independent and identically distributed (i.i.d.) initial conditions and their perturbations. However, for higher-dimension and more complex systems, the method requires a large number of samples to over-approximate accurately the reachable sets.

Over the years, the problem of computing the set of reachable sets of a given system has received considerable attention. In this section, we attempt to summarize this work and conclude with an argument for the novelty of the current manuscript. There are two main methods for reachability analysis: model-based and data-driven. Model-based reachability analysis uses a mathematical model of the system to compute reachable states from a given set of possible initial states. Over the years, several reachability tools have been developed, such as SpaceEx,^9^ JuliaReach,^10^ XSpeed,^11^ and Flow*,^8^ to name a few. The reachability methods have been widely used in applications that range from formal system verification to their synthesis.^4^ Reachability analysis is also at the core of abstraction-based techniques for controller synthesis in both deterministic systems^12,13^ and stochastic models.^14,15^

We will focus on data-driven reachability analysis techniques, which have also been proposed for scenarios when a model of the system is unavailable or too complex, and we will use the following axes to compare and position related papers, as summarized in Table 1.

**Table 1.** Positioning of the state of the art.

Paper	SUS	MSUS	IRS	IRU	G
16	H	M	PK	NT	NTG
21	H	M	PK	NT	NTG
20	H	M	FK	DI	NTG
23	H	M	PK	NT	PG
25	H	M	FK	NT	NTG
26	H^a^	M	FK	NT	NTG
28	NL	M	NK	NT	PG
29	NL	M	NK	NT	NTG
30	NL	M	NK	NT	NTG
31	NL	M	NK	NT	PG
32	NL	D	PK^b^	NT	FG
Our work	NL	*D*	*PK*	NT	PG

SUS: system-under-study; MSUS: modularity of SUS; IRS: information-required-from-SUS; IRU: information-required-from-user; PK: partial knowledge; NT: numerical tolerances; NTG: numerical tolerance guarantees; FK: full knowledge; DI: dynamic invariants; PG: probabilistic guarantees; NL: nonlinear; NK: no knowledge; FG: full guarantees.

a Restricted to two continuous modes.

b Submodels have to implement set-based reachability methods.

**Figure 1.**
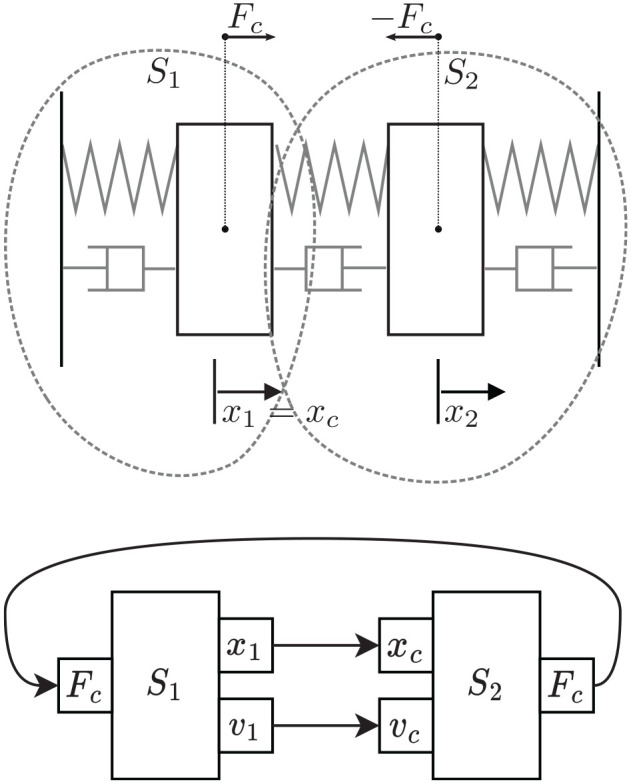
Example double mass-spring-damper system.

System-under-study (SUS): Denotes the kind of system supported by the technique. Systems can be linear/affine (*L*, the two are equivalent since one can transform an affine system into a linear one through extension of the states); nonlinear (*NL*, the type of Equation (1)); hybrid linear (*HL*, systems with different modes but within each mode the dynamics are linear); and hybrid (*H*, as in the most general hybrid automata). Within the hybrid category, there are kinds of systems, but we abstain from discerning those.
Modularity of SUS (MSUS): Represents the degree of support for decoupled SUS. The categories are monolithic (*M*) and decoupled (*D*). For example, systems that are represented by communicating sub-models, like the one presented in Figure 1, are decoupled.
Information-required-from-SUS (IRS): Denotes the degree of information that the technique requires from the SUS. Possible categories are full knowledge (*FK*) of the systems equations; partial knowledge (*PK*), where for example, the Jacobian of the system can be queried through an API, without the knowledge of the equations; and no knowledge (*NK*), where the model can be simulated through an API, without any knowledge of the equations.
Information-required-from-User (IRU): Denotes the kind of information the user needs to specify. At the very least, we have information on numerical tolerances (*NT*), and on the opposite side, we have information on dynamic invariants (*DI*).
Guarantees (G): Denotes the level of guarantees offered by the technique. We can have reachability up to numerical tolerance (*NTG*), probabilistic guarantees (*PG*), and guarantees including numerical approximations, that is, full guarantees (*FG*).

### 2.1. Dynamic sensitivity-based reachability analysis

We begin with the works that are based on solving or estimating the solution to the dynamic sensitivity equations, and then using their solution to build the reachable set, as introduced in Background and Problem Statement. Among these, we highlight the methods described in the study by Donzé and Maler,^16^ where a notion of expansion function is introduced, which can be seen as the application of the dynamic sensitivity to a given disturbance in the initial condition (cf. Theorems 3 and 4 in the study by Donzé and Maler^16^). The benefit of this method is that more simulations can be run, and in fact, thanks to the dynamic sensitivity information, the initial conditions can be iteratively tried in a way that attempts to drive the system into an unsafe state (to quickly falsify a safety property). In the same way, more samples can be taken, if more accuracy is needed. In the same paper, the technique was extended to hybrid systems without reset actions (but reset actions could be included, provided they are differentiable with respect to their inputs). The extension requires that the dynamic sensitivity of the jump time be computed as part of the system, and uses results developed earlier in, for example, the study by Hiskens and Pai.^17^ Later, Geng and Hiskens^18^ revisits the jump conditions required to apply second-order sensitivity analysis to hybrid systems (second-order sensitivity analysis permits an approximation of the flow around a nominal trajectory that will have an error in the order of $\epsilon^{3}$ ). The guarantees given are subject to the numerical approximation errors made by the underlying solver library, and on how fine-grained the sampling is, which is controlled by a tolerance parameter provided by the user. This method has been implemented into the Breach tool,^19^ and we classify it in Table 1 as requiring full knowledge from the system because of its hybrid systems extension. For nonlinear systems, only partial knowledge is required.

Another similar approach to sensitivity-based reachability analysis is proposed in C2E2,^20^ which originally was designed for continuous and switched systems and, in the later paper,^21^ extended to handle hybrid systems as well. Their work proposes a generic “discrepancy function,” which provides a time-varying maximum distance bound on any two trajectories originating from the initial set. As far as we could assess, the notions of a discrepancy function and an expansion function are closely related, with both capable of being generated from the dynamic sensitivity equations of the system, or over-approximations of it. The reader can see various methods for computing discrepancy functions for different classes of models in the study by Fan and Mitra,^22^ and the DryVR tool^23^ expresses the problem of finding a discrepancy function as a problem of learning a linear separator. The tool also provides a probabilistic accuracy guarantee on the computed discrepancy function, given a sampling complexity formula is followed.

HS^3^V^24,25^ is a similar tool, which uses sampling and a Lipschitz-based discrepancy function to estimate reachable sets. Their approach also introduces a method called dynamic simulations-spawning (s-spawning) to bound error growth and adds new simulations to deal with discrete jumps. It is worth mentioning a few other simulation-based approaches^26,27^ that provide methods to compute a time-varying function that provides a distance bound on trajectories between the system and a simpler counterpart. The simulations of the simpler model can be combined with the time-varying function to yield reachable sets.

#### 2.1.1. Optimization-based reachability analysis

The paper by Xue et al.^28^ uses samples obtained from simulating a black-box model to learn an underlying model by solving a robust optimization problem, which provides probabilistic model accuracy guarantees. Different template models can be used for learning the black-box model (e.g. polynomial functions). A similar approach is presented in work^29^ where the author’s approach uses sampled noisy data to identify a set of models, which are then over-approximated with zonotopes.

The paper^30^ presented a sampling-based reachability analysis approach that is based on random set theory and adversarial sampling. The main novelty of the work is utilizing recent advances in deep learning to iteratively discover trajectories that help to converge the actual reachable set. In other learning-based reachability analysis work, the NeuReach tool^31^ was introduced that efficiently computes reachable sets and provides a probabilistic accuracy guarantee.

While learning-based methods can improve the performance of the reachability analysis, the main drawback is that the underlying deep learning model has to be retrained for different systems.

#### 2.1.2. Decoupled reachability analysis

Finally, we highlight the work by Coënt et al.,^32^ which acknowledges the need for reachability analysis techniques that work in parallel for de-coupled models, such as those commonly found in co-simulation scenarios.^33^ In the aforementioned paper, the authors introduce an interval-based reachability method, which uses set-valued Runge–Kutta integration methods.^34^ The reachability computation is done step-by-step, advancing time after the reachable set of each step has been computed. At each step, each sub-model is a black-box simulation that computes the interval of outputs based on the interval of inputs. All sub-models’ intervals are then exchanged and the step is repeated until a fixed point is reached. A method for ensuring the robust stability of FMI co-simulation models has been presented in paper.^35^

### 2.2. Novelty of contribution

As summarized in Table 1, compared to the state of the art, the novelty of our contribution is in providing probabilistic guarantees for decoupled black-box models.

## 3. Background and problem statement

### 3.1. Continuous time systems

We consider continuous-time systems, characterized by a tuple $\Sigma =\left(X,x_{0},f\right)$ , where $X\subset R^{n}$ is the state space and n the number of states in the system, $x_{0}\in X$ represents the initial state, and f:X→X represents the vector field and is assumed to be locally Lipschitz continuous (any small changes in x result in bounded changes in f(x) ). The evolution of the state of Σ satisfies the following equation:

(1) $$\overset{\cdot }{x}\left(t\right)=f\left(t,\;x\left(t\right)\right),\;x\left(0\right)=x_{0},$$

which, thanks to the local Lipschitz assumption, always has a unique solution, regardless of the initial condition.

In order to represent the solution of Equation (1) as a function of time $t\in R_{\geq 0}$ , and the initial state $x_{0}\in X$ , we use the notation $\varphi (t,x_{0})\in X$ . For any finite simulation time $\tau \in R_{\geq 0}$ , and for all t∈[0,τ] , the continuous function $\varphi (t,x_{0})$ is a solution to Equation (1), and therefore satisfies the following equation:

(2) $$\overset{\cdot }{\varphi }\left(t,x_{0}\right)=f\left(t,\varphi \left(t,x_{0}\right)\right),$$

with $\varphi (0,x_{0})=x_{0}$ . Finally, note that $\varphi (t,x_{0})$ is continuous both in t and in $x_{0}$ .

### 3.2. Reachability analysis

Reachability analysis is a technique for computing the set of all reachable states of the solution to Equation (1) for each possible initial condition from a set $X_{0}\subseteq X$ . The reachable set $R_{t}$ at time t can be defined formally as follows:

(3) $$R_{t}\left(X_{0}\right)=\left\{\varphi \left(t,x_{0}\right)\;|\;x_{0}\in X_{0}\right\}$$

To capture all reachable states, starting from the initial time up to a given simulation time τ , we construct a flowpipe, which is just the union of all reachable states up to τ :

(4) $$R_{\left[0,\tau \right]}\left(X_{0}\right)=\underset{t\in \left[0,\tau \right]}{⋃}R_{t}\left(X_{0}\right)$$

Reachability methods provide a powerful approach to verifying safety requirements of dynamical systems under uncertainty,^4^ and are supported in a range of tools such as SpaceEx,^9^ Checkmate,^36^ and Flow*.^8^ Furthermore, to efficiently and accurately over-approximate reachable sets, different convex and nonconvex set representations have been developed. We refer the reader to the aforementioned works for more details on how to over-approximate the reachable set in Equation (4).

### 3.3. Co-simulation and the FMI standard

Co-simulation is a technique where multiple black-box simulators are coupled together (see the studies by Gomes et al.^33^ and Fitzgerald et al.^37^ for introductions to the topic). The difference between a black-box simulator and a black-box model is that the simulator contains the sub-model and approximates its numerical solution, given an input signal. Since simulators are coupled in feedback loops, the coupled solution is computed iteratively, moving forward in time and approximating the solution at each new time point from the solution at previous time steps. The FMI standard^38^ establishes the interface of the black-box simulators, also called FMUs, in the nomenclature of the standard. An individual FMU is comprised of a description file (in XML), which declares visible-state variables and other model information, and binaries that implement the application programming interface to interact with the FMU. Over the years, a number of well-known modeling and simulation tools have been upgraded (e.g. Simulink,^39^ OpenModelica^40^) or developed (INTO-CPS tool^41^) to support FMI standard.

The mandatory interface functions, implemented by an FMU denoted as S , are: doStep (*S*, *H*) (asks S to advance time to t+H and estimate internal state and outputs at the new time); setIn(*S, u, v*) (set the input of S identified by u to the value v for the current time t ); and getOut(*S*, *y*) (get the value for the output of S identified by y for the current time t ).

A co-simulation scenario is a set of FMUs and a description of how they are connected. It is often depicted in a diagrammatic form, as Example 1 shows.

**Example 1.** Consider the canonical example of a double mass-spring-damper system, depicted in Figure 1. The system is decoupled into two different FMUs, with inputs and outputs as depicted in the same figure. Then, with an interface similar to the FMI standard, their co-simulation is computed as illustrated in Algorithm 1.

**Table:** 

**Algorithm 1:** Example co-simulation orchestration for Example 1.
**Inputs:** A final simulation time $t_{f}>0$ , a communication step size H>0 , and FMUs $S_{1}$ and H>0 t←0 Initialize $S_{1}$ and $S_{2}$ **while t<t−f do** doStep ( $S_{1}$ , H ) doStep ( $S_{2}$ , H ) setIn ( $S_{1}$ , $F_{c}$ , getOut ( $S_{2}$ , $F_{c}$ )) setIn ( $S_{2}$ , $x_{c}$ , getOut ( $S_{1}$ , $x_{1}$ )) setIn ( $S_{2}$ , $v_{c}$ , getOut ( $S_{1}$ , $v_{1}$ )) t←t+H **end** **Output:** A value for each input/output computed at each time $t\in [0,t_{f}]$ .

FMU: functional mock-up unit.

In addition to the mandatory functions each FMU implements, the FMI also adds a number of optional functions, that can be optionally implemented by FMU exporting tools. From these, we highlight the functions that allow one to compute partial derivatives. Neglecting efficiency issues, we denote this function as getDer(*S, x, y*), which returns $\frac{\partial x}{\partial y}$ for the current time and state of s . These will be used later in the section “Building sensitivity equations co-simulation scenarios” to build the dynamic sensitivity equation system of a co-simulation scenario.

### 3.4. Problem statement

In this paper, we address the problem of computing reachable states of DT virtual models as formally defined in Problem 1.

**Problem 1.** Given a black-box Digital Twin model of a system Σ , initial set $X_{0}$ , and time-bound T , compute an approximation of the reachable set ${\bar{R}}_{\left[0,T\right]}(X_{0})$ using a finite number of randomly simulated trajectories of Σ . Provide the sample complexity of the computation, that is, the required number of trajectories for achieving a certain level of approximation with probabilistic confidence.

In the above problem statement, we assume that a black-box model of the system Σ is available, which can be used to generate sample trajectories from any initial state. These sample trajectories are sufficient for applying our first technique to solve the above problem. Our second technique requires also having access to trajectories of the dynamic sensitivity in the FMUs of the system.

### 3.5. Dynamic sensitivity equations

We define the dynamic sensitivity equations, also called the variational equations or just sensitivity equations, of the system in Equation (1) as the different derivatives of the n state variables with respect to the n initial conditions. For example, for a system with one dimension, the dynamic sensitivity equations represent how $\varphi (t,x_{0})$ changes as a function of changes in the initial condition $x_{0}$ . We represent this rate of change by the derivative $\frac{d\varphi (t,x_{0})}{dx_{0}}$ .

For a system with n dimensions, we will represent the state variable in each dimension i by $x_{i}$ , such that each state x∈X is represented by a vector $x=[x_{1},\ldots ,x_{n}{]}^{T}$ . Furthermore, we will represent the restriction of the solution $\varphi (t,x_{0})$ to the state variable $x_{i}$ as $\varphi_{i}(t,x_{0})$ , so that $\varphi (t,x_{0})={\left[\varphi_{1}(t,x_{0}),\ldots ,\varphi_{n}(t,x_{0})\right]}^{T}$ .

Given state variables $x_{i}$ and $x_{j}$ , we will use the shorthand notation $\delta_{i,j}(t,x_{0})$ to denote the derivative of $\varphi_{i}(t,x_{0})$ with respect to $x_{j,\mathrm{in}}$ (the initial value for $x_{j}$ ) as follows: $\delta_{i,j}\left(t,x_{0}\right)=\frac{\partial \varphi_{i}\left(t,x_{0}\right)}{\partial x_{j,\mathrm{in}}}$ .

The dynamic sensitivity is a matrix represented as follows:

(5) $$S\left(t,x_{0}\right)=\left[\begin{array}{ccc} \delta_{1,1}\left(t,x_{0}\right) & \ldots & \delta_{1,n}\left(t,x_{0}\right)\\ \vdots & \ddots & \vdots \\ \delta_{n,1}\left(t,x_{0}\right) & \ldots & \delta_{n,n}\left(t,x_{0}\right) \end{array}\right]$$

The dynamic sensitivity equations shown next represent an extension of Equation (1) with differential equations that relate $S(t,x_{0})$ to its time derivative $\overset{\cdot }{S}(t,x_{0})$ (derived below) as follows:

(6) $$\begin{array}{c} \overset{\cdot }{x}\left(t\right)=f\left(x\left(t\right)\right),\;\;x\left(0\right)=x_{0},\\ \overset{\cdot }{S}\left(t\right)=J\left(x\left(t\right)\right)\cdot S\left(t\right),\;\;S\left(0\right)=I, \end{array}$$

where we have omitted the dependency to $x_{0}$ of each solution to improve readability,· is the matrix product, and the following equation:

(7) $$J\left(x\left(t\right)\right)=\left[\begin{array}{ccc} \frac{\partial f_{1}\left(x\left(t\right)\right)}{\partial x_{1}} & \ldots & \frac{\partial f_{1}\left(x\left(t\right)\right)}{\partial x_{n}}\\ \vdots & \ddots & \vdots \\ \frac{\partial f_{n}\left(x\left(t\right)\right)}{\partial x_{1}} & \ldots & \frac{\partial f_{n}\left(x\left(t\right)\right)}{\partial x_{n}} \end{array}\right]$$

represents the Jacobian matrix of the continuous-time system and $\frac{\partial f_{i}(x(t))}{\partial x_{j}}$ denotes the partial derivative of the ith state derivative with respect to the jth state (recall that f is a vector function).

To derive Equation (6), we differentiate S(t) with respect to time. Each entry ${\overset{\cdot }{\delta }}_{i,j}(t,x_{0})$ of $\overset{\cdot }{S}(t)$ is therefore expanded as follows:

(7) $$\begin{array}{c} {\overset{\cdot }{\delta }}_{i,j}\left(t,x_{0}\right)=\frac{d}{dt}\frac{\partial }{\partial x_{j,\mathrm{in}}}\varphi_{i}\left(t,x_{0}\right)\;(\;\mathrm{expand}\;\mathrm{notation})\\ =\frac{\partial }{\partial x_{j,\mathrm{in}}}\frac{d}{dt}\varphi_{i}\left(t,x_{0}\right)\;\left(\mathrm{swap}\;\mathrm{derivative}\;\mathrm{order}\right)\\ =\frac{\partial }{\partial x_{j,\mathrm{in}}}f_{i}\left(t,\varphi \left(t,x_{0}\right)\right)\;\left(\mathrm{apply}\;\mathrm{Equation}\;(2)\right)\\ =\frac{df_{i}\left(t,\varphi \left(t,x_{0}\right)\right)}{dx}\cdot \frac{\partial \varphi \left(t,x_{0}\right)}{\partial x_{j,\mathrm{in}}}\;(\;\mathrm{apply}\;\mathrm{chain}\;\mathrm{rule})\\ =\underset{i\mathrm{th}\;\mathrm{row}\;\mathrm{of}\;J(x(t))}{\underset{︸}{\left[\begin{array}{ccc} \frac{\partial f_{i}\left(t,\varphi \left(t,x_{0}\right)\right)}{\partial x_{1}} & \ldots & \frac{\partial f_{i}\left(t,\varphi \left(t,x_{0}\right)\right)}{\partial x_{n}} \end{array}\right]}}\underset{j\mathrm{th}\;\mathrm{column}\;\mathrm{of}\;S(x(t))}{\underset{︸}{\left[\begin{array}{c} \frac{\partial \varphi_{1}\left(t,x_{0}\right)}{\partial x_{j,\mathrm{in}}}\\ \vdots \\ \frac{\partial \varphi_{n}\left(t,x_{0}\right)}{\partial x_{j,\mathrm{in}}} \end{array}\right]}} \end{array}$$

Taking all entries of $\overset{\cdot }{S}(t)$ together yields the equation $\overset{\cdot }{S}=J\cdot S$ . Note that each entry depends on the full state solution of the original system $\varphi (t,x_{0})$ and therefore the differential equation needs to be solved together with the original equations of the system. A system with n dimensions will therefore be extended to a system with $n+n^{2}$ dimensions.

**Figure 2.**
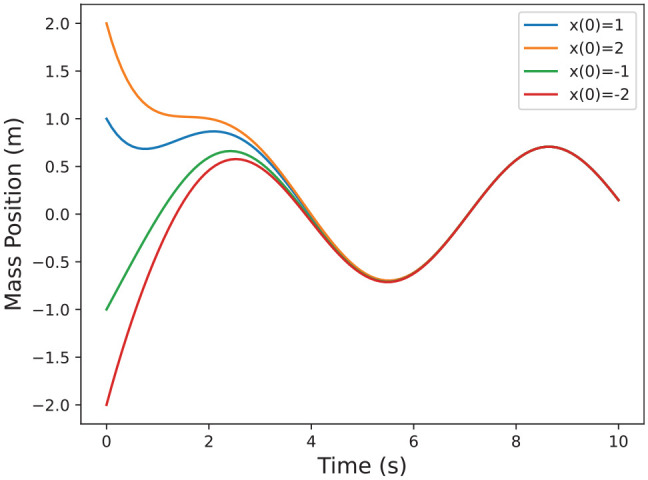
Example solutions for the system in Example 2.

**Example 2.** Consider the system given by the differential equation $\overset{\cdot }{x}=-x+\sin(t)\;x(0)=x_{0}$ , and its solution given by $\varphi (t,x_{0})=x_{0}e^{-t}+0.5\left(\sin(t)-\cos(t)+e^{-t}\right)$ (Taken from the study by Robinson^42^). The solution is plotted for different initial conditions in Figure 2. Since the initial conditions stop making a difference in the system (because of the periodic forcing function), we expect the sensitivity to vanish after about 6 s.

**Figure 3.**
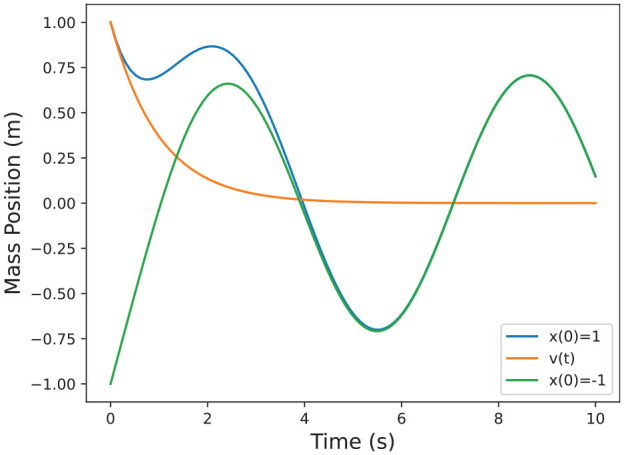
Example solutions for the system in Example 2 including the sensitivity.

Applying Equation (6), the expanded system is as follows:

(8) $$\begin{array}{c} \overset{\cdot }{x}=-x+\sin\left(t\right);\;x\left(0\right)=x_{0}\\ \overset{\cdot }{S}=-S;\;S\left(0\right)=1, \end{array}$$

with solution:

(9) $$\begin{array}{c} \varphi \left(t,x_{0}\right)=x_{0}e^{-t}+0.5\left(\sin\left(t\right)-\cos\left(t\right)+e^{-t}\right)\\ S\left(t\right)=e^{-t} \end{array}$$

plotted in Figure 3.

**Example 3.** Consider a spring pendulum whose behavior is given by the following dynamical system:

(10) $$\left[\begin{array}{c} \overset{\cdot }{r}\\ \overset{\cdot }{\theta }\\ {\overset{\cdot }{v}}_{r}\\ {\overset{\cdot }{v}}_{\theta } \end{array}\right]=\left[\begin{array}{c} v_{r}\\ v_{\theta }\\ {\mathrm{rv}}_{\theta }^{2}+9.8\cos\theta -2\left(r-1\right)\\ -\frac{2v_{r}v_{\theta }+9.8\sin\theta }{r} \end{array}\right]$$

The sensitivity matrix is therefore as follows:

(11) $$S\left(x\right)=\left[\begin{array}{cccc} \delta_{r,r} & \delta_{r,\theta } & \delta_{r,v_{r}} & \delta_{r,v_{\theta }}\\ \delta_{\theta ,r} & \delta_{\theta ,\theta } & \delta_{\theta ,v_{r}} & \delta_{\theta ,v_{\theta }}\\ \delta_{v_{r},r} & \delta_{v_{r},\theta } & \delta_{v_{r},v_{r}} & \delta_{v_{r},v_{\theta }}\\ \delta_{v_{\theta },r} & \delta_{v_{\theta },\theta } & \delta_{v_{\theta },v_{r}} & \delta_{v_{\theta },v_{\theta }} \end{array}\right]$$

As we show next, the Jacobian, J(x(t)) in Equation (6), of this system is as follows:

(12) $$J(x)=\left[\begin{array}{cccc} 0 & 0 & 1 & 0\\ 0 & 0 & 0 & 1\\ v_{\theta }^{2}-2 & -9.8\sin\theta & 0 & 2rv_{\theta }\\ \frac{2v_{r}v_{\theta }+9.8\sin\theta }{r^{2}} & -\frac{9.8}{r}\cos\theta & -\frac{2}{r}v_{\theta } & -\frac{2}{r}v_{r} \end{array}\right]$$

As we have seen before we can get the expression of $\overset{\cdot }{S}$ , the time derivative of the dynamic sensitivity matrix, using $\overset{\cdot }{S}=J\cdot S$ . We get the following 16 equations, which depend on the original system equations in Equation (10):

(12) $$\begin{array}{c} {\overset{\cdot }{\delta }}_{r,r}=\delta_{v_{r},r},{\overset{\cdot }{\delta }}_{r,\theta }=\delta_{v_{r},\theta },\;{\overset{\cdot }{\delta }}_{r,v_{r}}=\delta_{v_{r},v_{r}},\;{\overset{\cdot }{\delta }}_{r,v_{\theta }}=\delta_{v_{r},v_{\theta }}\\ {\overset{\cdot }{\delta }}_{\theta ,r}=\delta_{v_{\theta },r},\;{\overset{\cdot }{\delta }}_{\theta ,\theta }=\delta_{v_{\theta },\theta },\;{\overset{\cdot }{\delta }}_{\theta ,v_{r}}=\delta_{v_{\theta },v_{r}},\;{\overset{\cdot }{\delta }}_{\theta ,v_{\theta }}=\delta_{v_{\theta },v_{\theta }}\\ {\overset{\cdot }{\delta }}_{v_{r},r}=\left(v_{\theta }^{2}-2\right)\delta_{r,r}-9.8\sin\theta \delta_{\theta ,r}+2rv_{\theta }\delta_{v_{\theta },r}\\ {\overset{\cdot }{\delta }}_{v_{r},\theta }=\left(v_{\theta }^{2}-2\right)\delta_{r,\theta }-9.8\sin\theta \delta_{\theta ,\theta }+2rv_{\theta }\delta_{v_{\theta },\theta }\\ {\overset{\cdot }{\delta }}_{v_{r},v_{r}}=\left(v_{\theta }^{2}-2\right)\delta_{r,v_{r}}-9.8\sin\theta \delta_{\theta ,v_{r}}+2rv_{\theta }\delta_{v_{\theta },v_{r}}\\ {\overset{\cdot }{\delta }}_{v_{r},v_{\theta }}=\left(v_{\theta }^{2}-2\right)\delta_{r,v_{\theta }}-9.8\sin\theta \delta_{\theta ,v_{\theta }}+2rv_{\theta }\delta_{v_{\theta },v_{\theta }}\\ {\overset{\cdot }{\delta }}_{v_{\theta },r}=C\left(r,\theta \right)\delta_{r,r}-K\left(r,\theta \right)\delta_{\theta ,r}-\frac{2}{r}v_{\theta }\delta_{v_{r},r}-\frac{2}{r}v_{r}\delta_{v_{\theta },r}\\ {\overset{\cdot }{\delta }}_{v_{\theta },\theta }=C\left(r,\theta \right)\delta_{r,\theta }-K\left(r,\theta \right)\delta_{\theta ,\theta }-\frac{2}{r}v_{\theta }\delta_{v_{r},\theta }-\frac{2}{r}v_{r}\delta_{v_{\theta },\theta }\\ {\overset{\cdot }{\delta }}_{v_{\theta },v_{r}}=C\left(r,\theta \right)\delta_{r,v_{r}}-K\left(r,\theta \right)\delta_{\theta ,v_{r}}-\frac{2}{r}v_{\theta }\delta_{v_{r},v_{r}}-\frac{2}{r}v_{r}\delta_{v_{\theta },v_{r}}\\ {\overset{\cdot }{\delta }}_{v_{\theta },v_{\theta }}=C\left(r,\theta \right)\delta_{r,v_{\theta }}-K\left(r,\theta \right)\delta_{\theta ,v_{\theta }}-\frac{2}{r}v_{\theta }\delta_{v_{r},v_{\theta }}-\frac{2}{r}v_{r}\delta_{v_{\theta },v_{\theta }} \end{array}$$

where:

(12) $$\begin{array}{cccc} C\left(r,\theta \right) & =\frac{2v_{r}v_{\theta }+9.8\sin\theta }{r^{2}}, & K\left(r,\theta \right) & =\frac{9.8}{r}\cos\theta \end{array}$$

### 3.6. Interpretation of sensitivity equations

We demonstrate here how dynamic sensitivity equations can be used to approximate the reachable set $R_{\left[0,\tau \right]}(X_{0})$ in Equation (4). First note how the distance between the system solutions in Figure 3 for Example 2 is correlated to the sensitivity solution. Since $\varphi (t,x_{0})$ is a continuous function of $x_{0}$ , we can perform a Taylor expansion around the value $x_{0}$ as follows:

(13) $$\varphi \left(t,x_{0}+\epsilon \right)\approx \varphi \left(t,x_{0}\right)+\underset{S\left(t,x_{0}\right)}{\underset{︸}{\frac{d\varphi \left(t,x_{0}\right)}{dx_{0}}}}\epsilon +O\left(\epsilon^{2}\right)$$

where the $O(\epsilon^{2})$ denotes the order of the magnitude for the higher order terms in the rest of the Taylor series. Equation (13) gives us a direct method to estimate trajectories around a nominal system solution $\varphi (t,x_{0})$ . Note that the truncated terms are expected to be in the order of $\epsilon^{2}$ , which will be small in comparison with the first two terms of the Taylor expansion for small values of ϵ .

**Figure 4.**
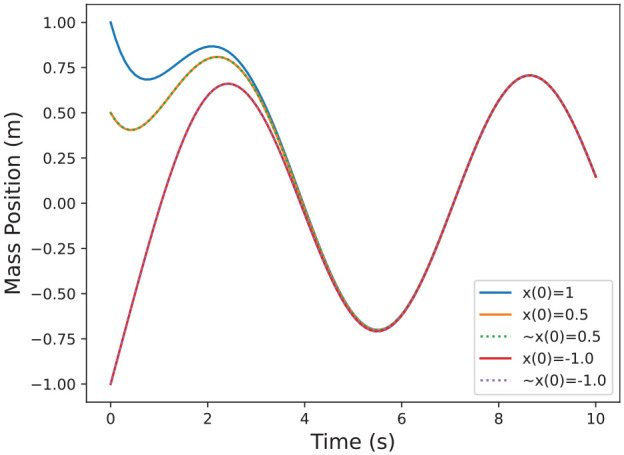
Example estimated solutions for the system in Example 2 around nominal trajectory φ(t,1) , as detailed in Example 4.

**Example 4.** Following Example 2, we know $S(t)=e^{-t}$ , so we can use it to estimate other trajectories around φ(t,1) . The result is plotted in Figure 4 where the dotted trajectories represent estimates, and the solid represent the actual solutions. Note that there is no error in the estimates because the system is linear, and therefore the higher order terms in Equation (13) vanish.

To summarize, for an expanded dynamic sensitivity system as in Equation (6), and a given initial set $X_{0}$ of potential initial conditions, the reachable set $R_{t}(X_{0})$ in Equation (3) can be approximated using the following procedure:

1. Discretize $X_{0}$ into smaller hyper-rectangles $X_{1},\ldots ,X_{n}$ such that the distance between any point contained in each hypercube and its center is small enough (generally smaller than 1 because of the truncated term in Equation (13)).
2. For each $X_{j}$ , compute the nominal solution at its center, and apply Equation (13) to estimate all trajectories of interest in its vicinity (for linear and affine systems, it suffices to cover all the extremities of $X_{j}$ ).
3. Because of continuity, any set of states between a trajectory and the estimated trajectories in its vicinity are reachable, so we can form flow pipes uniting the nominal trajectory and all trajectories of interest in its vicinity.
4.
$R_{t}(X_{0})$ is then computed by the union of all flow pipes.

The above approach does not necessarily generate over-approximations of the reach set for nonlinear systems since the higher-order terms in the Taylor expansion are eliminated without appropriate quantification of the induced error. In the following sections, we provide two techniques that are based on random trajectories of the system and provide probabilistic correctness guarantees.

### 3.7. Robust convex programs

This section provides the mathematical details for robust convex programs (RCPs) and data-driven approximations of their solution. The content of this section is provided in its full generality. We will utilize Theorem 1 and Theorem 2 presented in the sequel to establish the correctness of our data-driven framework. The reader can refer to the papers^43,44^ for the full exposition of the results presented in this section.

Let $T\subset R^{q}$ be a compact convex set for some q∈N and $c\in R^{q}$ be a constant vector. Let D be the space of uncertainty with (D,B,P) denoting the uncertainty probability space ( B is the Borel sigma-algebra on D and P a probability measure that assigns probabilities to sets in B ). Let g:T×D→R be a measurable function, which is convex in the first argument for each d∈D , and bounded in the second argument for each θ∈T . The *RCP* is defined as follows:

(14) $$\mathrm{RCP}:\left\{ \begin{array}{c} \min_{\theta }c^{T}\theta \\ s.t.\theta \in T\;\mathrm{and}g\left(\theta ,d\right)\leq 0;\;\forall d\in D \end{array}\right.$$

An example of the RCP used in our work is presented in Equation (23). Computationally tractable approximations of the optimal solution of the RCP given by Equation (14) can be obtained using scenario convex programs (SCP) that only require gathering finitely many samples from the uncertainty space.^44^

Let $(d_{i}{)}_{i=1}^{N}$ be N i.i.d. samples drawn according to the probability measure P . The SCP corresponding to the RCP given by Equation (14) strengthened with γ≥0 is defined as follows:

(15) $$\mathrm{SC}P_{\gamma }:\left\{ \begin{array}{c} \min_{\theta }\;c^{T}\theta \\ s.t.\;\theta \in T,\;\mathrm{and}\\ g\left(\theta ,d_{i}\right)+\gamma \leq 0;\;\forall i\in \{1,2,\ldots ,N\} \end{array}\right.$$

An example of the SCP used in our work is presented in Equation (24). We denote the optimal solution of RCP given by Equation (14) as $\theta_{\mathrm{RCP}}^{*}$ and the optimal solution of $\mathrm{SC}P_{\gamma }$ given by Equation (15) as $\theta_{\mathrm{SCP}}^{*}$ . Note that $\theta_{\mathrm{RCP}}^{*}$ is a single deterministic quantity but $\theta_{\mathrm{SCP}}^{*}$ is a random quantity that depends on the i.i.d. samples $(d_{i}{)}_{i=1}^{N}$ drawn according to P . The RCP given by Equation (14) is a challenging optimization problem since the cardinality of D is infinite and therefore the optimization has an infinite number of constraints. In contrast, the SCP given by Equation (15) is a convex optimization with a finite number of constraints for which efficient optimization techniques are available. The following two theorems provide sample complexity results for connecting the optimal solutions of the $\mathrm{SC}P_{\gamma }$ to that of the RCP .

**Theorem 1.** Let β∈(0,1) be a confidence value and ϵ∈(0,1) a given tolerance.^43^ Select the number of samples N according to:

(16) $$N\geq \frac{1}{\epsilon }\left(\frac{e}{e-1}\right)\log\left(\frac{1}{\beta }+q\right)$$

where e is Euler number and q is the dimension of the decision vector θ∈T . Then the solution of Equation (15) with γ=0 computed by taking N i.i.d. samples $(d_{i}{)}_{i=1}^{N}$ from P is a feasible solution for the constraint:

(17) P(g(θ,d)≤0)≥1−ϵ

with confidence (1−β) .

The above theorem states that if we take the number of samples appropriately, we can guarantee that the solution satisfies the robust constraint in Equation (14) on all the domain d∈D except for a small subset that has measure at most ϵ .

**Theorem 2.** Assume that the function g:T×D→R d→g(θ,d) in Equation (14) is Lipschitz continuous with respect to d∈D uniformly in θ∈T with Lipschitz constant $L_{d}$ and let $h:[0,1]\to R_{\geq 0}$ be a strictly increasing function such that:^44^

(18) $$P\left(\Omega_{\epsilon }\left(d\right)\right)\geq h\left(\epsilon \right),$$

for every d∈D and ε∈[0,1] . Let $\theta_{\mathrm{RCP}}^{*}$ be the optimal solution of the RCP in Equation (14) and $\theta_{\mathrm{SCP}}^{*}$ the optimal solution of $\mathrm{SC}P_{\gamma }$ in Equation (15) with:

(19) $$\gamma =L_{d}h^{-1}\left(\varepsilon \right)$$

computed by taking N i.i.d. samples $(d_{i}{)}_{i=1}^{N}$ from P . Then $\theta_{\mathrm{SCP}}^{*}$ is a feasible solution for the RCP with confidence (1−β) if the number of samples is at least N(ε,β) , where:

(20) $$N\left(\varepsilon ,\beta \right):=\min\left\{N\in N|\sum_{i=0}^{q-1}\left(\begin{array}{c} N\\ i \end{array}\right)\varepsilon^{i}{\left(1-\varepsilon \right)}^{N-i}\leq \beta \right\},$$

with q being the dimension of the decision vector θ∈T .

The above theorem is stronger than Theorem 1 in guaranteeing that the solution will be feasible for the RCP in Equation (14) on the whole domain d∈D . This is at the cost of requiring the knowledge of an upper bound on the Lipschitz constant of the function g and also being more conservative in the required number of samples. The confidence (1−β) is a common feature of these two theorems and is due to the nature of the solution that depends on the sampled dataset $(d_{i}{)}_{i=1}^{N}$ .

## 4. Reachability algorithms

In this section, we describe two different algorithms for computing reachable states of black-box FMI models. The two algorithms compute a scaling factor S , which is then used to compute edges of the reachable set as follows:

(20) $$\varsigma \left(t,x_{c}\right)\pm S\left(t\right)\|\eta /2{\|}_{\infty }$$

where $\varsigma (t,x_{c})$ denotes a central trajectory and η denotes the size of the discretized initial state-space. This section also describes a curve-fitting approach for estimating an upper boundary of the scaling factor and a method for building up the sensitivity matrix from the FMI’s dependency graph.

The first reachability algorithm uses simulated trajectories of a black-box model and *SCP* to compute a maximum Lipschitz constant of the black-box model. The computed Lipschitz constant together with a central trajectory is then used for computing an interval-based approximation of the reachable set. The alternative algorithm replaces the estimation of the model’s Lipschitz constant in the previous algorithm with a solution of sensitivity equations, which describe the impact of perturbations of the system’s initial conditions on the trajectories of the system.

These algorithms are presented in detail in the following sections.

### 4.1. Sampling-based algorithm

For computing the reachable set from a set of initial states $X_{0}$ , a common approach is to partition the set $X_{0}$ into a union of hyper-rectangles $\left\{X_{j},\;j=1,2,\ldots ,m\right\}$ of size $\eta =[\eta_{1},\eta_{2},\ldots ,\eta_{n}]$ by gridding the state space. Then for each $X_{j}$ , we find a vector $L_{j}(t)\in R^{n}$ such that:

(21) $$\begin{array}{c} \left|\varsigma \left(t,x_{0}\right)-\varsigma \left(t,{x\prime }_{0}\right)\right|\;\leq L_{j}\left(t\right)\|x_{0}-{x\prime }_{0}{\|}_{\infty }\\ \;\forall x_{0},\;{x\prime }_{0}\in X_{j},\;t\geq 0 \end{array}$$

where $\varsigma (t,x_{0})$ and $\varsigma (t,x\prime_{0})$ are the state trajectories of the system at time t started from $x_{0},x\prime_{0}\in X_{j}$ , and|·| denotes the element-wise absolute value. In the next step, the reachable set from each $X_{j}$ is computed as the hyper-rectangle $Y_{j}$ with edges as follows:

(22) $$\varsigma \left(t,x_{cj}\right)\pm L_{j}\left(t\right){\left\|\eta /2\right\|}_{\infty }$$

which gives a hyper-rectangle with center $\varsigma (t,x_{\mathrm{cj}})$ and size $L_{j}(t)\cdot \eta$ . The state $x_{\mathrm{cj}}$ is the center of the initial hyper-rectangle $X_{j}$ . The union of all $Y_{j}$ , j=1,2,…,m gives an over-approximation of the reachable set from $X_{0}$ . The implementation of the above procedure requires computing $\varsigma (t,x_{\mathrm{cj}})$ , which is possible using a black-box model of the system.

#### 4.1.1. RCP formulation and sampling

The inequality Equation (21) used in the reachability analysis can written as the RCP:

(23) $$\mathrm{RCP}:\left\{ \begin{array}{c} \min c^{T}L_{j}\left(t\right)\\ s.t.\;c=\left[1;1;\ldots ;1\right],L_{j}(t)\geq 0,\;\mathrm{and}\\ \left|\varsigma \left(t,x_{0}\right)-\varsigma \left(t,x_{\mathrm{cj}}\right)\right|-L_{j}\left(t\right)\|x_{0}-x_{\mathrm{cj}}{\|}_{\infty }\leq 0,\\ \forall x_{0}\in X_{j}. \end{array}\right.$$

We can define the associated $\mathrm{SC}P_{\gamma }$ :

(24) $$\mathrm{SC}P_{\gamma }:\left\{ \begin{array}{c} \min c^{T}L_{j}\left(t\right)\\ s.t.\;c=\left[1;\ldots ;1\right],L_{j}\left(t\right)\geq 0,\;\forall i\in \left\{1,\ldots ,N\right\},\\ |\varsigma \left(t,x_{0i}\right)-\varsigma \left(t,x_{\mathrm{cj}}\right)|-L_{j}\left(t\right)\|x_{0i}-x_{\mathrm{cj}}{\|}_{\infty }+\gamma \leq 0, \end{array}\right.$$

where $x_{0i}\in X_{j}$ are taken randomly from a probability distribution P .

Once the $\mathrm{SC}P_{\gamma }$ in Equation (24) is solved, the sampling-based reachable set from $X_{j}$ is computed as the hyper-rectangle ${\tilde{Y}}_{j}$ with edges $\varsigma (t,x_{\mathrm{cj}})\pm L_{j}(t)\|\eta /2{\|}_{\infty }$ where $L_{j}(t)$ is obtained by solving Equation (24). The next theorem uses the results of the section “Robust convex programs” for picking the number of samples N to connect ${\tilde{Y}}_{j}$ with the true reachable set.

**Theorem 3.** If ${\tilde{Y}}_{j}$ is computed using the solution of Equation (24) with γ=0 and N selected according to Equation (16), then with confidence (1−β) , the set ${\tilde{Y}}_{j}$ covers the whole true reachable set except for a small set with probability measure at most ϵ .

If ${\tilde{Y}}_{j}$ is computed using the solution of Equation (24) with N selected according to Equation (20), then with confidence (1−β) , the set ${\tilde{Y}}_{j}$ covers the whole true reachable set.

The full algorithm for our sampling-based reachability analysis is presented in Algorithm 2.

**Table:** 

**Algorithm 2:** Sampling-based reach set computation
**Inputs:** System as a black box, time instance t , initial set $X_{0}\subset R^{n}$ Select discretization $\eta =\left[\eta_{1},\eta_{2},\ldots ,\eta_{n}\right]$ with $\eta_{i}>0$ Partition $X_{0}$ into hyper-rectangles $X_{j}$ , j=1,2,…,m , of size η with center $x_{\mathrm{cj}}$ **for** j=1,2,…,m **do** Select N according to Equations (16) or (20) Take N samples $x_{0i}$ uniformly from $X_{j}$ Obtain trajectories $\varsigma (t,x_{0i})$ and $\varsigma (t,x_{\mathrm{cj}})$ from the black box model Solve the $\mathrm{SC}P_{\gamma }$ in Equation (24) to find $L_{j}(t)$ Define ${\tilde{Y}}_{j}$ as a hyper-rectangle with center $\varsigma (t,x_{\mathrm{cj}})$ and size $L_{j}(t)\|\eta /2{\|}_{\infty }$ **end** **Output:** Sampling-based reach set $\tilde{Y}:=\cup_{j}{\tilde{Y}}_{j}$

### 4.2. Lipschitz constant via extreme value theorem

For estimating $L_{d}$ in Theorem 2 and making use of it in Theorem 3, we should estimate an upper bound for the fraction:

(25) $$\Delta \left(x,x\prime \right):=\frac{∥\varsigma \left(t,x\right)-\varsigma \left(t,x\prime \right)∥}{∥x-x\prime ∥}$$

that holds for all $x,\;x\prime \in X_{j}$ . We follow the line of reasoning in the studies by Weng et al.^45^ and Wood and Zhang^46^ and use the extreme value theorem for the estimation.

Let us fix a δ>0 and assign uniform distribution to the pair (x,x′) over the domain $\left\{x,x\prime \in X_{j},∥x-x\prime ∥\leq \delta \right\}$ . Then Δ(x,x′) is a random variable with an unknown cumulative distribution function (CDF). Based on the assumption of Lipschitz continuity of the system, the support of the distribution of Δ(x,x′) is bounded from above, and we want to estimate an upper bound for its support. We take n samples from (x,x′) and compute n samples $\Delta_{1},\Delta_{2},\ldots ,\Delta_{n}$ for Δ(x,x′) . The CDF of $\max\left\{\Delta_{1},\Delta_{2},\ldots ,\Delta_{n}\right\}$ is called the limit distribution of Δ(x,x′) . The Fisher–Tippett–Gnedenko theorem says that if the limit distribution exists, it can only belong to one of the three families of extreme value distributions—the Gumbel class, the Fréchet class, and the Reverse Weibull class. These CDFs have the following forms:

(25) $$\mathrm{Gumbel}\left(\mathrm{Type}I\right):G\left(s\right)=\exp\left(-\exp\left(\frac{s-a}{b}\right)\right)$$

where s∈R :

Fréchet $\begin{array}{c} \left(\mathrm{Type}\mathrm{II}\right):G\left(s\right)=\left\{ \begin{array}{c} 0\;\mathrm{if}s<a\\ \exp\left(-{\left(\frac{s-a}{b}\right)}^{-c}\right)\;\mathrm{if}s\leq a \end{array}\right. \end{array}$

Rvr.Weibull $\begin{array}{c} \left(\mathrm{Type}\mathrm{III}\right):\;G\left(s\right)=\left\{ \begin{array}{c} \exp\left(-{\left(\frac{a-s}{b}\right)}^{c}\right)\;\mathrm{if}s<a\\ 1\;\mathrm{if}s\leq a \end{array}\right. \end{array}$

where a∈R,b>0,c>0 are, respectively, the location, scale, and shape parameters.

Among the above three distributions, only the Reverse Weibull class has support bounded from above. Therefore, the limit distribution of Δ(x,x′) will be from this class and the location parameter a is such an upper bound. As a result, we can estimate the location parameter of the limit distribution of Δ(x,x′) to get an estimation of the Lipschitz constant.

A procedure for estimating the Lipschitz constant is presented in Algorithm 3. This uses obtained Lipschitz constants to compute approximate reachable sets. For each state of the system, a single Lipschitz constant value is obtained from a previously sampled set. In this work, we considered two operations for obtaining a final $L_{s}\;(x,\;t)$ : a maximum value and a value produced via curve-fitting and the extreme value theorem.^47^ The algorithm then computes a central trajectory of the model by simulating it from the set of initial values which are midway between the lower and upper limits of the initial set.

**Table:** 

**Algorithm 3:** Lipschitz constant estimation using Reverse Weibull distribution
**Inputs:** System as a black box, time instance t , initial set $X_{j}\subset R^{n}$ Parameters: δ>0 , number of samples n,m **for** k=1,2,…,m **do** Take n samples $\left(x_{i},\;x\prime_{i}\right)$ uniformly from the set $\left\{x,x\prime \in X_{j},∥x-x\prime ∥\leq \delta \right\}$ Compute $\left\{\Delta \;(x_{i},{x\prime }_{i}),\;i=1,2,\ldots ,n\right\}$ using Equation (25) and trajectories from the black-box model Define $L_{k}=\underset{i}{\max}\;\Delta (x_{i},\;x\prime_{i})$ **end** Fit a Reverse Weibull distribution to the dataset $\left\{L_{1},L_{2},\ldots ,L_{m}\right\}$ Get the location, scale and shape parameters of the fitted distribution **Output:** Estimated Lipschitz constant as the location parameter of the fitted distribution

**Remark**. The estimated Lipschitz constant from Algorithm 3 can also be used directly for estimating the reachable sets. Unfortunately, this quantity is just an estimation and will converge to the true Lipschitz constant in the limit. When it is computed with a finite number of samples, it is not associated with a quantitative closeness guarantee. In contrast, using the vector $L_{j}(t)$ for reachability computations is more likely to give less conservative reach sets with formal probabilistic closeness guarantees.

### 4.3. Sensitivity-based algorithm

In this section, we describe an alternative algorithm, which uses solutions of dynamic sensitivity equations to replace scaling of the initial region with a Lipschitz constant factor $L_{j}(t)$ , with rescaling based on the sensitivity matrix $S(x_{\mathrm{in}},\;t)$ .

The algorithm similarly partitions initial region $X_{0}$ into a union of hyper-rectangles $\left\{X_{j},\;j=1,2,\ldots ,m\right\}$ of size $\eta =[\eta_{1},\eta_{2},\ldots ,\eta_{n}]$ . The algorithm then requires obtaining a system of sensitivity equations $\overset{\cdot }{S}(t)$ and solving them numerically together with black-box system $\overset{\cdot }{x}(t)$ from an N number of randomly sampled initial conditions $x_{0i}$ within each hyper-rectangle $X_{j}$ .

The reachability algorithm then over-approximates the image $Y_{j}$ of the hyper-rectangle $X_{j}$ , by first computing expansion vectors:

(25) $$\xi^{i}=[\xi_{1}^{i},\ldots ,\xi_{n}^{i}]\;\mathrm{where}\xi_{k}^{i}=\left|S(t,x_{0i})\right|\cdot (\eta {/2)}^{T}$$

which use the sensitivity matrix $S(t,x_{0i})$ (or rather, its element-wise absolute value $\left|S(t,x_{0i})\right|$ ) to compute the maximum expansion in each direction of the sample point $x_{0i}$ . The method then takes the element-wise maximum $\xi^{\max}=[\overset{N}{\underset{i=1}{\max}}\;\xi_{1}^{i},\ldots ,\overset{N}{\underset{i=1}{\max}}\;\xi_{n}^{i}]$ , which is used to compute the edges of $Y_{j}$ by expanding around the central trajectory $\varsigma (t,x_{\mathrm{cj}})$ .

The full algorithm is described in Algorithm 4.

**Table:** 

**Algorithm 4:** Sensitivity-based reach set computation
**Inputs:** Time instance t , initial set $X_{0}\subset R^{n}$ Select discretization $\eta =\left[\eta_{1},\eta_{2},\ldots ,\eta_{n}\right]$ with $\eta_{i}>0$ Partition $X_{0}$ into hyper-rectangles $X_{j}$ , j=1,2,…,m , of size η with center $x_{\mathrm{cj}}$ Acquire system of dynamic sensitivity equations $\overset{\cdot }{S}(t)$ **for** j=1,2,…,m **do** Select N according to Equations (16) or (20) Take N samples $x_{0i}$ uniformly from $X_{j}$ Obtain central trajectory $\varsigma (t,x_{\mathrm{cj}})$ and sensitivity matrix $S(t,x_{0i})$ from the black-box model Compute expansion vectors $\xi^{i}=[\xi_{1}^{i},\ldots ,\xi_{n}^{i}]$ where $\xi_{k}^{i}=\left|S(t,x_{0i})\right|\cdot (\eta /2{)}^{T}$ Compute maximum expansion vector $\xi^{\max}=[\overset{N}{\underset{i=1}{\max}}\;\xi_{1}^{i},\ldots ,\overset{N}{\underset{i=1}{\max}}\;\xi_{n}^{i}]$ Define ${\tilde{Y}}_{j}$ as a hyper-rectangle with center $\varsigma (t,x_{\mathrm{cj}})$ and size $\xi^{\max}$ **end** **Output:** Sampling-based reach set $\tilde{Y}:=\cup_{j}{\tilde{Y}}_{j}$

### 4.4. Building sensitivity equations co-simulation scenarios

In this section, we describe how to extend Algorithm 4 to handle networks of FMUs implementing the FMI interface.

**Figure 5.**
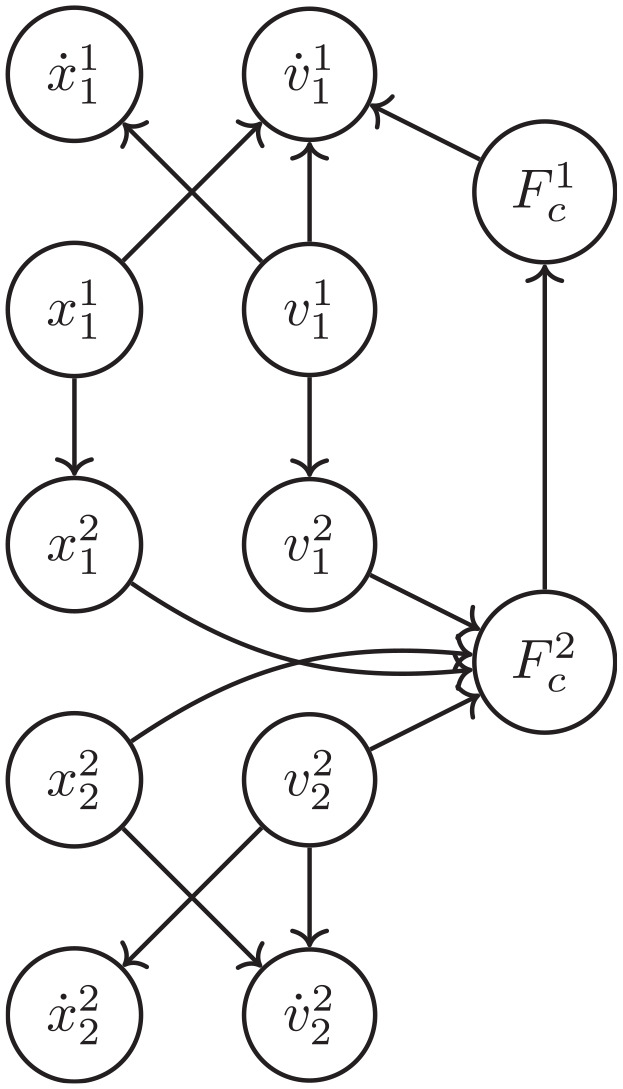
The dependency graph example of the mass spring damper example.

Given the network structure of FMUs, in order to get the Jacobian matrix required to compute the sensitivity matrix, we need a way to differentiate a variable in one FMU with respect to a variable in another FMU (recall Equation (6)). For that reason, we build a dependency graph before the sampling starts. The vertex of this graph are the state variables of each FMU, their time derivatives, and the input and output variables. The edges represent the dependency of the target on the source. For example, given the system and implementation in Figure 1, its dependency graph is depicted in Figure 5.

**Remark**. If a dependency graph has a cycle, a variable depends on itself. This is not a typical behavior of systems in the form of Equation (1) and is therefore outside the scope of this paper.

We can use the dependency graph to know what computations we need to do in order to calculate a derivative, as follows. Given variables α and β , let D denote all cycle-free paths from α to β . The derivative of α with respect to β is as follows:

(26) $$\frac{d\alpha }{d\beta }=\sum_{p\in D}\overset{|p|-2}{\underset{i=0}{\Pi }}\frac{\partial p\left[i+1\right]}{\partial p\left[i\right]},$$

where, given path p,|p| denotes its length and p[n] denotes the nth element of p .

For example, in Figure 5, $\frac{d{\overset{\cdot }{v}}_{1}^{1}}{{\mathrm{dx}}_{1}^{1}}$ is given as follows. There are two paths: $x_{1}^{1}\to {\overset{\cdot }{v}}_{1}^{1}$ and $x_{1}^{1}\to x_{1}^{2}\to F_{c}^{2}\to F_{c}^{1}\to {\overset{\cdot }{v}}_{1}^{1}$ . Hence:

(27) $$\begin{array}{c} \frac{d{\overset{\cdot }{v}}_{1}^{1}}{dx_{1}^{1}}=\frac{\partial {\overset{\cdot }{v}}_{1}^{1}}{\partial x_{1}^{1}}\;(1\mathrm{st}\mathrm{path})\\ +\frac{\partial x_{1}^{2}}{\partial x_{1}^{1}}\frac{\partial F_{c}^{2}}{\partial x_{1}^{2}}\frac{\partial F_{c}^{1}}{\partial F_{c}^{2}}\frac{\partial {\overset{\cdot }{v}}_{1}^{1}}{\partial F_{c}^{1}}\;(2\mathrm{nd}\mathrm{path}) \end{array}$$

In order to compute the sensitivity matrix, we initialize it to an identity matrix of the correct dimension. After that, each sample step is a co-simulation run, where we compute the Jacobian at every co-simulation step, calling a function that computes every partial derivative that makes an element of the Jacobian matrix J(x(t)) using Equation (26). Once we have the Jacobian for time t , we estimate the dynamic sensitivity matrix using a numerical solver. For simplicity, we use the Forward Euler method: $S(t+H)=S(t)+\overset{\cdot }{S}(t)*H=S(t)+J(x(t))\cdot S(t)*H$ , where $\overset{\cdot }{S}(t)$ is computed as in Equation (6) and H is the co-simulation step-size parameter. We provide a formalized summary of the algorithms in Algorithm 5.

**Table:** 

**Algorithm 5:** Compute the sensitivity matrix of a system in the FMI standard
**Input:** A set of FMUs FS and their inter-connections, the communication step size H , the final simulation time $t_{f}>0$ . Initialize S and J to the identity matrix t←0 **while** $t<t_{f}$ **do** Exchange data among all FMUs Compute J using Equation (26) **for all F∈FS do** doStep (F,H) **end** S←S+J·S*H t←t+H **end** **Output:** The dynamic sensitivity matrix S after an arbitrary number of steps.

FMI: functional mock-up interface; FMU: functional mock-up unit.

## 5. Validation experiments

This section presents validation exercises that evaluate our reachability algorithms as presented in the previous section. The validation exercises cover both affine dynamical systems and nonlinear systems and aim to evaluate the conservativeness of the computed reachable sets and the associated computation time. We also obtain reachable sets (and computation time) produced by the model-based reachability tool Flow* and compare them against ones produced by our methods. To select nonlinear system benchmarks and Flow* parameters, we followed a well-known verification competition ARCH.^48^

### 5.1. Experiment setup

All timing results in this section were measured on an HP EliteBook 840 G7 with an Intel Core i5-10310U processor under Ubuntu 22.04 (Linux 5.14.0). For the methods described in this paper, the results are based on a prototype implementation in Python. In particular, we relied on the SciPy^49^solve_ivp function and the LSODA solver^50^ for solving dynamical systems (with an absolute tolerance parameter of atol = 10^−6^ and a relative tolerance parameter rtol = 10^−3^), while SCP optimization problems were solved via the CVXPY library^51,52^ with the parameter γ=0 . Comparison results and timings for Flow* were produced by Flow* toolbox.

#### 5.1.1. Affine systems

We can start to evaluate the performance of our method on Linear/Affine Initial Value Problems of form:

(28) $$\frac{d}{dt}x\left(t\right)=Ax\left(t\right)+b;\;x\left(0\right)\in x_{0}$$

with state matrix $A\in R^{n\times n}$ and offset vector $b\in R^{n}$ , and interval vector initial region $x_{0}\in IR^{n}$ . While linear systems pose a significantly easier reachability challenge than general nonlinear systems—in this case, sensitivity analysis is exact, while Flow* and SpaceEx both provide very efficient special-purpose reachability algorithms—they allow us to effectively evaluate how well the methods of this paper approximate a given linear system’s dynamics, since these are well understood and admit explicit solutions.

**Figure 6.**
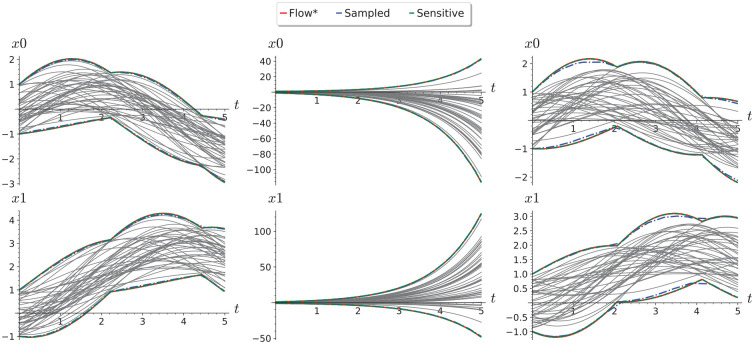
Comparison of reachability from sampled Lipschitz constants with Flow* and Sensitivity Analysis results for a randomly generated 2D stable system (left), an unstable system (middle), and an oscillator (right) from the unit initial region [−1, 1]^2^. Numerical simulations (gray) for 100 randomly sampled initial conditions are shown for comparison.

Sample reachability results for different classes of linear systems are shown in Figure 6. We can see that Flow* and sensitivity-based reachability analysis both produce indistinguishable flowpipes, while applying reachability analysis based on the Lipschitz constant computed from sampled trajectories alone gives a coarser reachable set estimation shown.

### 5.2. Lipschitz constant estimation accuracy

To assess the overall accuracy of our methods, we will consider uniformly randomly selected N -dimensional Affine Systems of the form Equation (28), restricted such that $A\in [-1,\;1{]}^{N}$ , b∈[−1,1] , and $x_{0}\subseteq [-1,\;1{]}^{N}$ . We will consider separately the classes of stable systems (those for which every eigenvalue of A has a negative real part) and unstable systems (those for at least one eigenvalue of A has a positive real part), and take 100 systems of each class.

We will assess how accurately each of the different methods captures the dynamics of the underlying system based on the vector of Lipschitz constants, which they use to compute reachable sets. While the SCP optimization directly computes a vector $L_{\mathrm{SCP}}(t)$ of Lipschitz constants for the system, we are also able to compute a similar vector of Lipschitz constants from the sensitivity matrix as $L_{\mathrm{sens}}(t)\overset{\Delta }{=}c\left|S(t)\right|$ where c=[1,…,1] and |M| is the element-wise absolute value of the matrix M . and considering the accuracy of each method to estimate the Lipschitz constant of the system over-approximate the system dynamics. We will compare each of these approximations to the true vector of Lipschitz constants (with respect to $\|\cdot {\|}_{\infty }$ ) for the system, which we can compute using the general solution of a linear ODE as, L(t)=c|exp(At)|. Then we may measure the relative absolute error of an approximated Lipschitz constant vector L′(t) at a given time point t as follows:

(28) $$\mathrm{RAE}\overset{\Delta }{=}\frac{{\left\|L\prime \left(t\right)-L\left(t\right)\right\|}_{2}}{{\left\|L\left(t\right)\right\|}_{2}}.$$

Then, we may estimate the overall performance by taking the geometric mean relative absolute error (GMRAE; The geometric mean is preferred over the mean when aggregating error rates due to the latter’s sensitivity to outliers,^53^ such as those arising from numerical errors when computing the RAE of small quantities.) of multiple sampled relative absolute errors $\mathrm{RA}E_{i}$ via the formula:

(28) $$\mathrm{GMRAE}\overset{\Delta }{=}{\left(\overset{n}{\underset{i=1}{\Pi }}\mathrm{RA}E_{i}\right)}^{\frac{1}{n}}.$$

**Figure 7.**
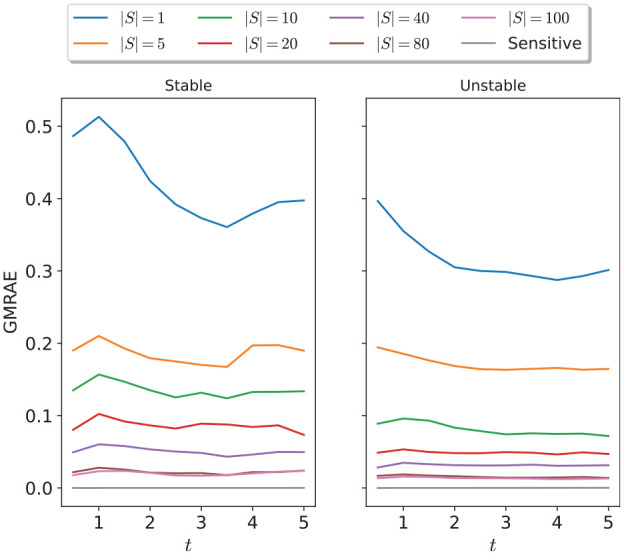
Errors of different methods of Lipschitz estimation at different time points between 1.0 and 5.0 for stable and unstable random 2D linear systems.

**Figure 8.**
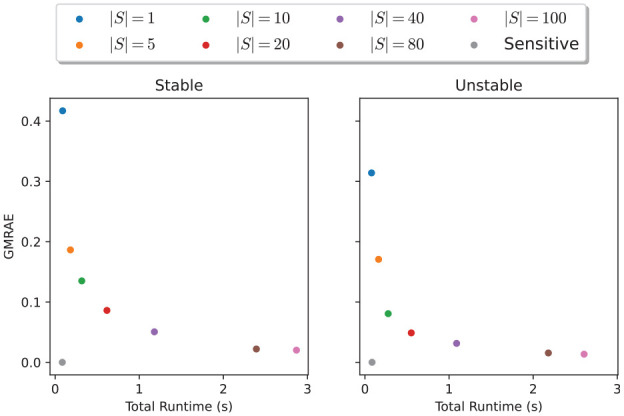
Comparison of total runtime against GMRAE for stable and unstable random 2D linear systems.

In the special case of two-dimensional (2D) systems, Figure 7 shows the evolution of the GMRAE of the Lipschitz constant vector estimate produced using dynamical sensitivity analysis, and SCP optimization for varying numbers of samples. We see that the relative error from SCP optimization decreases with an increasing number of samples, and is roughly consistent over the whole simulation time. In addition, the relative error of the method is similar between stable and unstable systems; this result is somewhat surprising given that typical Lipschitz constants for random unstable systems can be orders of magnitude larger than those of stable systems (and, indeed, the absolute error of the method will be correspondingly larger for the same number of samples). Figure 8 shows the trade-off between the total runtime of each method and the relative error achieved. We observed a relationship between the number of samples and the relative error improvement in the relative error trailing off after 80 samples. Finally, we observed that, as expected, dynamical sensitivity analysis (with a single sampled sensitivity matrix) approximates the true Lipschitz constant vector almost perfectly for linear systems, and provides by far the best accuracy/runtime trade-off for 2D systems.

**Figure 9.**
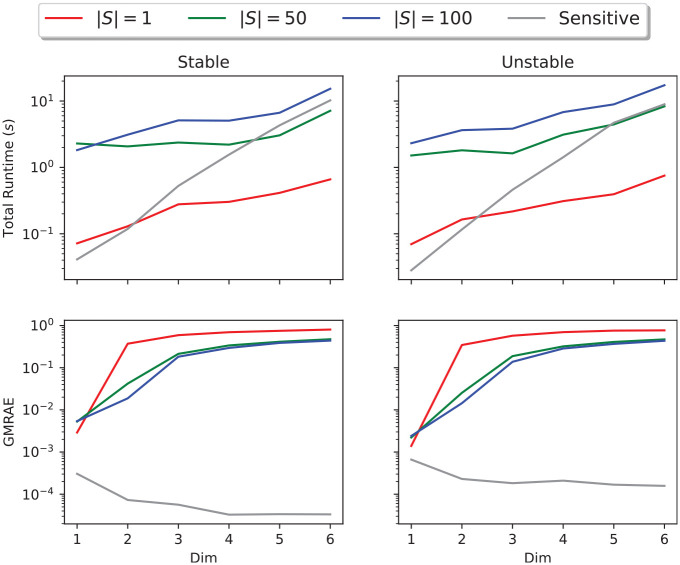
Errors and runtimes of different methods of Lipschitz estimation at time for t=5.0 randomly sampled linear systems of up to six dimensions.

In addition, Figure 9 shows how the runtime and relative error of each method varies with the dimension of the system, based on 100 randomly sampled stable and unstable system for dimensions 1 through 6. We can see that the runtime of each method increases exponentially with the system dimension and that the rate of increase of sampling runtime increases with the number of samples, while the runtime of dynamic sensitivity analysis increases significantly more rapidly than the SCP optimization-based approximation with any of these numbers of samples. However, dynamic sensitivity consistently produced the best approximation of the system Lipschitz constant vector, and indeed, its relative error decreased with the dimension of the system. This suggests that the dynamic sensitivity equations are a reliable method of estimating the Lipschitz constant of linear systems, with consistent accuracy regardless of system dimension, while sampling offers a flexible cost/accuracy trade-off for higher-dimensional systems.

#### 5.2.1. Nonlinear systems

This section compares our proposed algorithms for computing reachable sets and validates them against a model-based reachability analysis tool—Flow*. Let us start by considering 2D nonlinear Van Der Pol system as follows:

(29) $$\left\{ \begin{array}{c} \overset{\cdot }{x}\left(t\right)=y\left(t\right)\\ \overset{\cdot }{y}\left(t\right)=\left(1-x{\left(t\right)}^{2}\right)\cdot y-x \end{array}\right.$$

**Figure 10.**
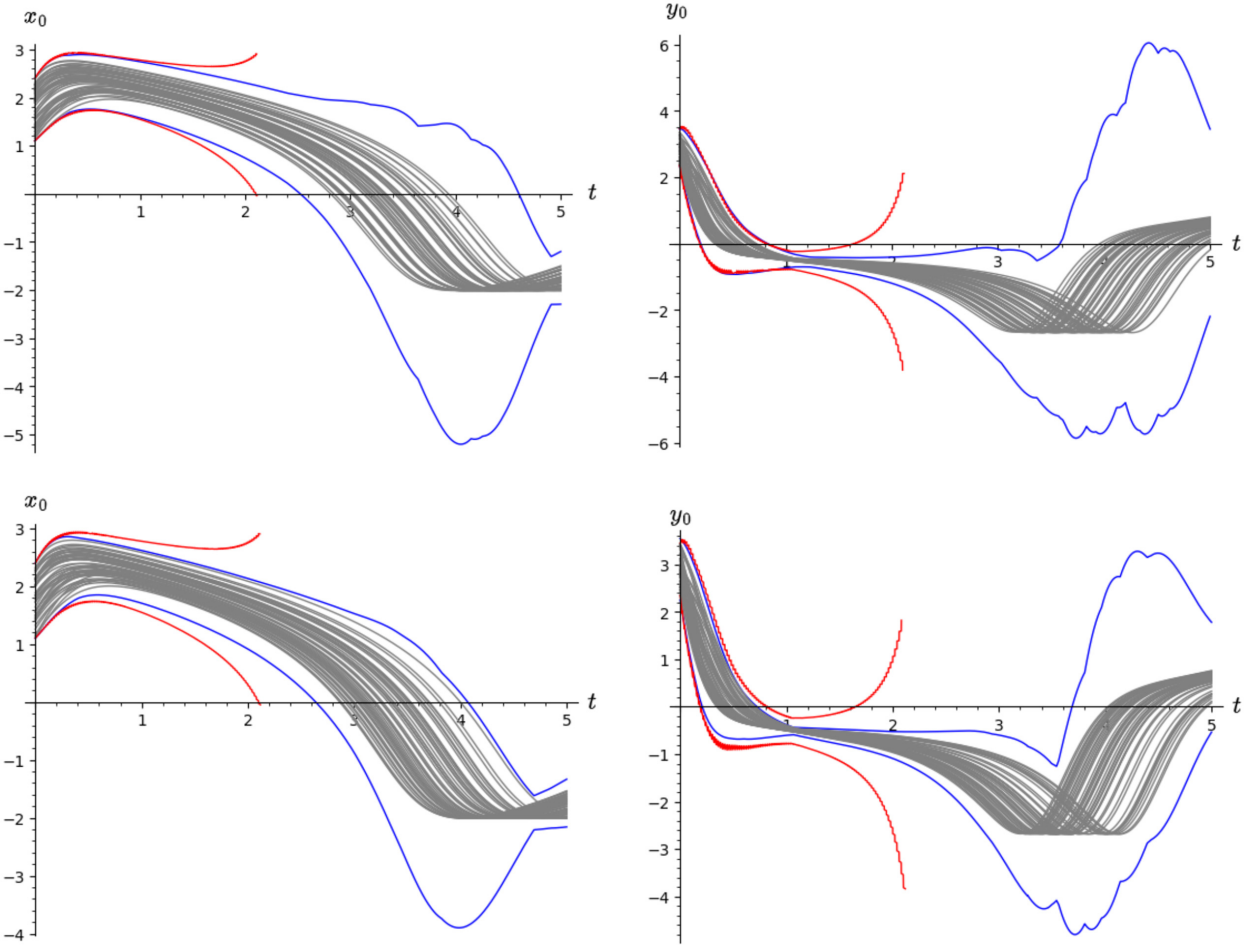
Reachable set comparison of the nonlinear Van Der Pol system for the initial set [1.1, 2.4] × [2.35, 3.45] for T=[0,5] . **Top:** reachable set produced by a sensitivity-based algorithm for x state (left) and y state (right), **Bottom:** reachable sets produced by a sampling-based algorithm for x state (left) and y state (right). Both algorithms were used with 100 samples.

Figure 10 compares the reachable set for the initial set [1.1, 2.4] × [2.35, 3.45] computed using by Algorithms 2–4 and Flow*. The top figures show reachable sets produced by sensitivity-based Algorithm 4 (blue curve), Flow* (red curve) and some randomly sampled trajectories (gray curves) for x,y states of the Van Der Pol system respectively. Flow* was not able to produce reachable sets over the whole time horizon [0,5] with the given initial region.

**Figure 11.**
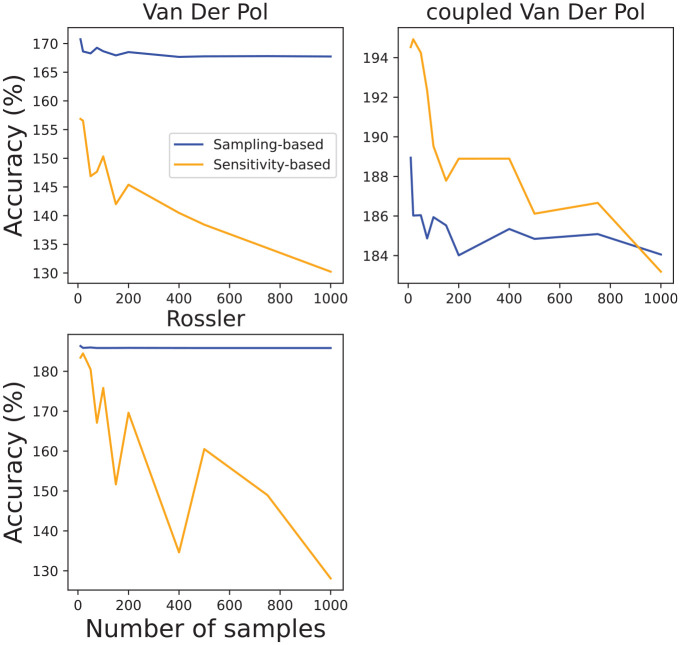
Volume error exercise that demonstrates the number of samples effects on volume accuracy. We consider the following number of samples [10, 20, 50, 75, 100, 150, 200, 400, 500, 750, 1000].

The rest of the section considers four additional nonlinear models with varying number of dimensions: coupled Van Der Pol (four-dimensional (4D)), Rossler System (three-dimensional (3D)), Spring Pendulum (4D, model from the Dynamic Sensitivity Equations section) and Biological Model (seven-dimensional (7D)). We evaluate the runtime and flowpipe volume accuracy produced by Algorithms 2 and 4. The latter is measured by using Equation (30) as follows:

(30) $$A=\sum_{t=0}^{T}\left(100-\left(\frac{V\mathrm{ol}\left(R_{S}\left(t\right)\right)-V\mathrm{ol}\left(R_{F}\left(t\right)\right)}{V\mathrm{ol}\left(R_{F}\left(t\right)\right)}\times 100\right)\right)$$

where $V\mathrm{ol}(R_{S}(t))$ and $V\mathrm{ol}(R_{F}(t))$ are volumes of reachable sets produced, respectively, by one of our algorithms and Flow* at time t with δ size step. The metric measures an accumulated proportional volumetric difference between two flowpipes (e.g. negative A would indicate that in comparison to Flow* one of our algorithms produces a less conservative flowpipe). From Figure 11, we can observe that the sampling-based algorithm computes a more conservative flowpipes, however, this comes at a cost of requiring more samples, hence computation time, to guarantee an over-approximation, especially for larger initial regions.

**Figure 12.**
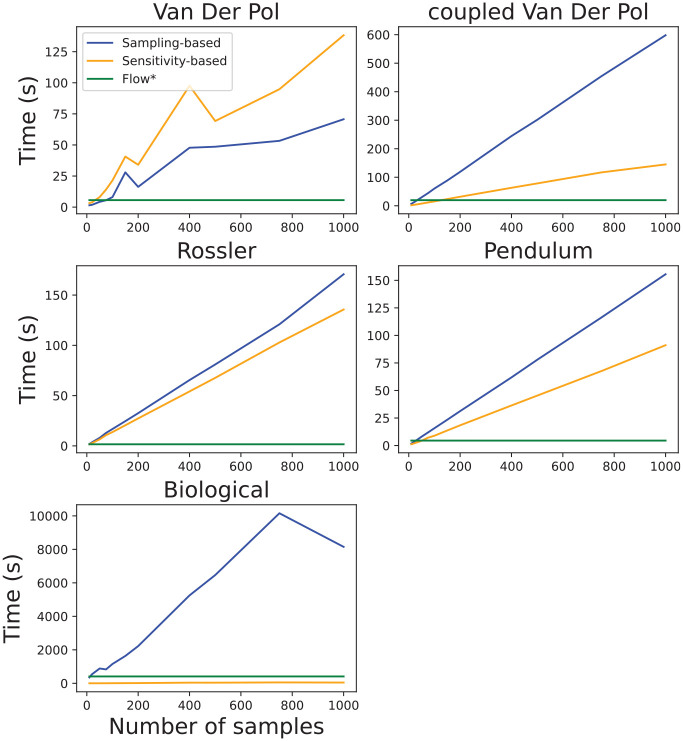
Runtime validation exercise that demonstrates the number of samples effects to computation time of reachable sets for different nonlinear models.

Similar findings can be observed from Figure 12 in which we summarize our accuracy results from three models for different number of samples: Van Der Pol initial state: $x_{1}=[1.1,\;1.4]$ , $y_{1}=[2.35,\;2.45]$ , coupled Van Der Pol parameters $x_{1,2}=[1.25,\;1.55]$ , $y_{1,2}=[2.35,\;2.45]$ , T=[0,5] , while Rossler system x=[0.7,1] and y,z=1 ; all systems analyzed for [0,5] s. We decided to exclude results from Spring Pendulum and Biological models as Flow* was only able to produce reachable sets from small initial sets and for short time horizons, resulting in minuscule flowpipe volumes.

The runtime validation experiments are summarized in Figure 12. In these experiments, we again increased the number of samples for Algorithms 2 and 4 and observed reachable set computation time. We also include the runtime performance of the Flow* tool. Important to note that at this stage, we did not attempt to improve the computational performance of the proposed methods.

**Figure 13.**
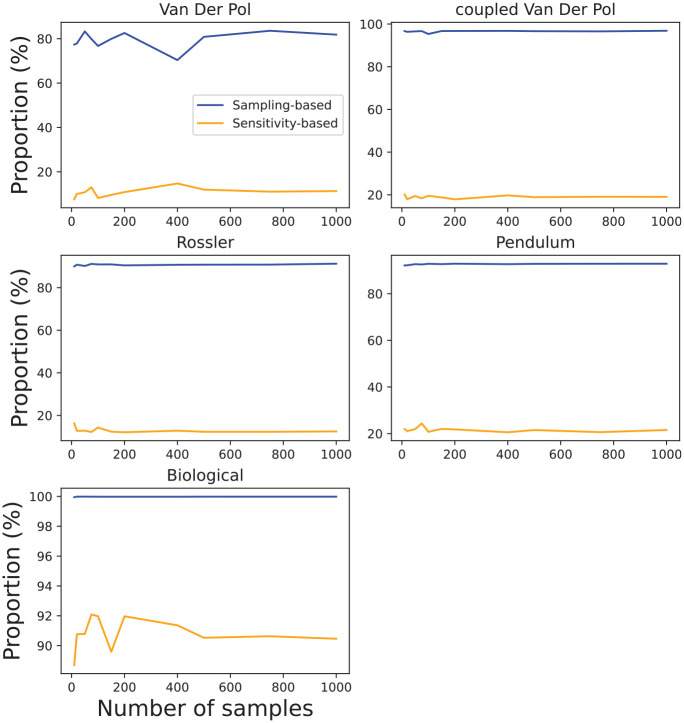
The proportion of runtime in Figure 12 to perform sampling and solving SCP in Algorithm 2, and sample and solve sensitivity equations in Algorithm 4. The rest of the runtime is used for computing flowpipe.

Figure 12 clearly shows that Algorithm 2 is considerably slower in comparison to Algorithm 4 and does not scale well with an increased number of samples. The main reason for this is the computation overhead of solving SCPs . We can see this in Figure 13 in which we demonstrate the proportion of runtime it takes to sample and solve the SCP in Algorithm 2 and solve sensitivity equations in Algorithm 4 for different models and numbers of samples. Except for the case of the Biological model, solving sensitivity equations in Algorithm 4 makes up a significantly smaller proportion of computation time, while the opposite is true in the case of obtaining maximum Lipschitz constant with SCPs in Algorithm 2.

In short, the results presented in this section have shown that our algorithms produce reasonably conservative reachable sets for nonlinear systems. Although, with the current algorithm implementation, their runtimes do not scale well with the increased number of samples, we have shown accurate results can be produced even with a fairly small number of samples. The main limitation of the Algorithm 2 is the need for a larger number of samples to provide probabilistic accuracy guarantees, while solving SCP is a major contributor to a large runtime. The sensitivity-based algorithm provides much less conservative results but offers a more scalable approach.

#### 5.2.2. Sensitivity matrix co-simulation

In order to validate the results of the algorithms given in “Building sensitivity equations co-simulation scenarios,” we are going to use the mass spring damper system visualized in Figure 1. The equations that describe this system’s behavior are provided in Equation (31):

(31) $$\left[\begin{array}{c} {\overset{\cdot }{x}}_{1}\\ {\overset{\cdot }{v}}_{1}\\ {\overset{\cdot }{x}}_{2}\\ {\overset{\cdot }{v}}_{2}\\ F_{c} \end{array}\right]=\left[\begin{array}{c} v_{1}\\ \frac{-c_{1}\cdot x_{1}-d_{1}\cdot v_{1}+F_{c}}{m_{1}}\\ v_{2}\\ \frac{-c_{2}\cdot x_{2}-F_{2}}{m_{2}}\\ c_{c}\cdot \left(x_{2}-x_{1}\right)+d_{c}\cdot \left(v_{2}-v_{1}\right) \end{array}\right]$$

**Figure 14.**
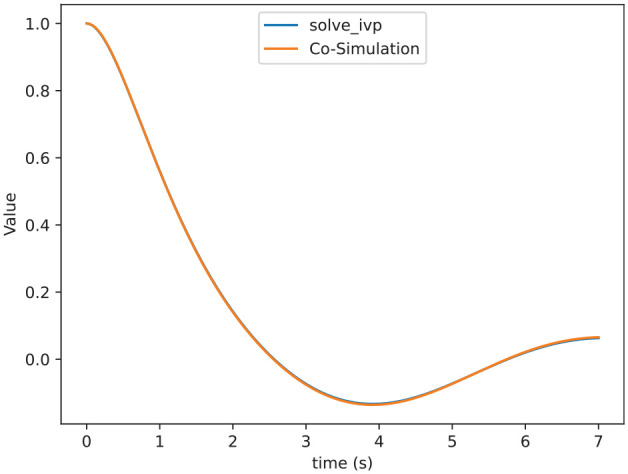
Comparison of $\delta_{x_{1},x_{1}}$ computed by Algorithm 5 and the solve_ivp ODE solver.

**Figure 15.**
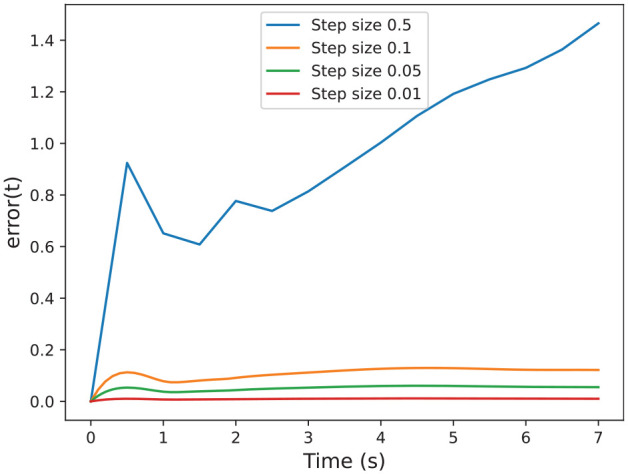
Errors between the sensitivity matrix computed by Algorithm 5 and solve_ivp method with varying time steps sizes.

We are going to solve this system together with the coupled sensitivity equations using the SciPy solve ivp solver. In Figure 14, we validate the value of $\delta_{x_{1},x_{1}}$ (an element of the sensitivity matrix computed with Algorithm 5) against the analytical solution, with a time step of 0.01 . We will then compute the error between the sensitivity matrix computed by Algorithm 5 and the solve ivp solver function as follows:

(32) $$e\left(t\right)=\|S\left(t\right)-S\prime \left(t\right){\|}_{2}$$

where S(t) denotes the sensitivity matrix computed by the Algorithm 5, S′(t) denotes the sensitivity matrix computed by the solve_ivp function, and $∥\cdot {∥}_{2}$ denotes the 2-norm for matrices. In Figure 15, we show different error functions for different step sizes, which shows that the smaller the step size, the smaller the error.

We can see in the results that our approximation is close enough to the solve_ivp function. Figure 14 shows that both functions are almost indistinguishable. Furthermore, Figure 15 shows that by decreasing the step size of the co-simulation scenario we can reduce the error, which allows us to get as close as we want to standard numerical algorithms.

#### 5.2.3. Validation discussion

In this section, we explored the ability of each of our methods to accurately and efficiently approximate the dynamics of black-box models and to conservatively compute reachable sets.

First, we saw that in the case of linear systems, the sampling-based approach is able to approximate the sensitivity of the system to its initial conditions (as captured in the vector of Lipschitz constants), and the accuracy of this approximation can be increased by increasing the number of samples. This is consistent with Theorem 3, which specifies the number of samples required to achieve a given probability of over-approximating the true Lipschitz constants and, consequently, the true reachable set. We also saw that for linear systems, dynamic sensitivity analysis gives an almost exact approximation of the true Lipschitz constants regardless of system dimension, although its runtime increases rapidly with the dimension of the system.

For nonlinear systems, both sampling and dynamic sensitivity analysis give approximate results, while their conservativeness can both be increased by increasing the number of samples used. For most of our systems, we saw that dynamic sensitivity analysis gives reasonable results for a reasonably low runtime. However, the sampling-based approach is able to give more conservative results for higher numbers of samples and is also able to give probabilistic guarantees on containment.

We also saw how we can do sensitivity analysis to decoupled FMUs, by dynamically tracking the sensitivity matrix of the system. This is limited by the fact that our current co-simulation technique relies on the Forward Euler method, which produces larger errors than more competitive numerical integration methods. The use of better numerical methods is important to reduce these errors, but this would impose additional requirements on the FMUs being simulated. In practice, we observed relatively small errors between the sensitivity matrices computed via this method and the conventional open-box method using the LSODA solver.

## 6. Conclusion and future work

Ensuring the dependability of DTs relies on proving that the formal system models underpinning them are safe. In some cases, accurate models of complex systems are too difficult to obtain or unavailable due to IP protection (as facilitated by the FMI standard). In this work, we develop methods to provide formal analysis for models featuring uncertainty or unavailability of their dynamics, by introducing algorithms for performing reachability analysis of black-box models. We were particularly focused on the FMI standard-based black box dynamical system models. The developed data-driven and dynamic sensitivity–based reachable set computation methods have been thoroughly evaluated for linear and nonlinear dynamical systems, and results have shown that conservative reachable sets can be computed. Although, as discussed, for large numbers of samples and high-dimensional systems, the runtime performance of the algorithms offers scope for improvement (particularly the sampling-based algorithm), we saw that algorithms do not require a large number of samples to produce accurate reachable sets.

There are several interesting directions for future work:

1. We could investigate extending each of the methods proposed in this paper from reachability analysis, to monitoring Signal Temporal Logic properties of the system’s behavior following the methodology of Wright and Stark.^54^ This would allow us to verify whether black box models satisfy high-level temporal logic specifications, while accounting for the impact of uncertainty on the result of verification via three-valued logic and probabilistic guarantees.
2. We could investigate the application of each of the methods to parametric black-box models, as a way to soundly account for the impact of uncertain model parameters on the behavior of the system.
3. Our sampling-based approach can in general be applied to hybrid models as long as trajectories are continuous functions of the initial state. To apply the dynamic sensitivity–based approach to hybrid automata, we would like to investigate how dynamic sensitivity equations could be obtained for a black-box hybrid system.
4. In our future work, we also aim to explore the integration of our proposed method with the DT system and replace simulations with data obtained from the physical asset. A similar approach has been presented in work by Van Acker et al.^55^
